# Can AMSTAR also be applied to systematic reviews of non-randomized studies?

**DOI:** 10.1186/1756-0500-7-609

**Published:** 2014-09-06

**Authors:** Dawid Pieper, Tim Mathes, Michaela Eikermann

**Affiliations:** Institute for Research in Operative Medicine, Witten/Herdecke University, Ostmerheimer Str. 200, Building 38, D-51109 Cologne, Germany

**Keywords:** Systematic review, Psychometrics, Evidence synthesis, Observer agreement

## Abstract

**Background:**

There is a lack of an instrument to evaluate systematic reviews of non-randomized studies in epidemiological research. The Assessment of Multiple Systematic Reviews (AMSTAR) is widely used to evaluate the scientific quality of systematic reviews, but it has not been validated for SRs of non-randomized studies. The objective of this paper is to report our experience in applying AMSTAR to systematic reviews of non-randomized studies in terms of applicability, reliability and feasibility. Thus, we applied AMSTAR to a recently published review of 32 systematic reviews of non-randomized studies investigating the hospital volume-outcome relationship in surgery.

**Results:**

The inter-rater reliability was high (0.76), albeit items 8 (scientific quality used in formulating conclusions), 9 (appropriate method to combine studies), and 11 (conflicts of interest) scored moderate (≤0.58). However, there was a high heterogeneity between the two pairs of reviewers. In terms of feasibility, AMSTAR proved easy to apply to systematic reviews of non-randomized studies, each review taking 5–10 minutes to complete. We faced problems in applying three items, mainly related to scientific quality of the included studies.

**Conclusions:**

AMSTAR showed good psychometric properties, comparable to prior findings in systematic reviews of randomized controlled trials. AMSTAR can be applied to systematic reviews of non-randomized studies, although there are some item specific issues users should be aware of. Revisions and extensions of AMSTAR might be helpful.

## Background

Systematic reviews (SRs) are the cornerstone of evidence-based health care. They can provide the highest level of evidence [1, 2]. Following this follows that conducting methodological sound SRs is a crucial point for health care professionals and researchers. Much focus has been put on the critical appraisal of primary studies which is a major part in an evidence synthesis. However, not only the critical appraisal of primary studies is important, but also the critical appraisal of SRs itself is important in order to ensure a solid basis for decision making. Over the years, many tools have been developed to assess the methodological quality of SRs. The Overview Quality Assessment Questionnaire (OQAQ) [3, 4] and Assessment of Multiple Systematic Reviews (AMSTAR) [5–7] are two widely used tools for the assessment of systematic reviews. Two surveys of overviews (systematic reviews of reviews) found both instruments to be used frequently in this context [8, 9].

It has to been acknowledged that AMSTAR has been developed upon the OQAQ and the checklist by Sacks [10] and can therefore be seen as the most recent tool, being introduced in 2007. It consists of 11 items and was found to be valid, reliable and easy to use [11]. According to the developers, AMSTAR can be applied to a wide variety of SRs, although it is recognized that it has only been tested on SRs of randomized controlled trials evaluating treatment interventions [7].

However, it is well-known that RCTs are not feasible for a wide range of research questions where we have to rely on evidence from non-randomized studies (NRS) instead. While investigating the hospital volume-outcome relationship in surgery, we conducted an overview (review of reviews) due to the huge amount of literature published in this research area [12]. It is known that the vast majority of studies investigating this relationship are observational. Furthermore, volume is usually treated as a continuous variable, while volume categories are often constructed for the statistical analysis. This means that we are mainly not investigating interventions, but risk factors (defined as distinct volume categories). To the best of our knowledge there was no assessment tool for SRs of NRS available at the time of our work, so we decided to apply AMSTAR to all included SRs, although AMSTAR was originally not developed and tested for this purpose.

The objective of this paper is to report our experience and challenges in applying AMSTAR to SRs of risk factors in NRS in terms of applicability. Furthermore, we also aimed to investigate the reliability and feasibility.

## Methods

We used a recently published systematic review of systematic reviews investigating the volume-outcome relationship in surgery that was conducted by our research team. Details of the methods have been reported elsewhere [12]. In brief, we searched several databases for systematic reviews investigating the relationship between high-volume hospitals and outcomes in surgery. We included 32 SRs. Twenty six SRs focused on a specific procedure while the remaining 6 SRs had no specific focus and included several procedures. The methodological quality of each SR was assessed independently with the AMSTAR tool by two reviewers. In total, there were three reviewers, one reviewer assessed all SRs. The other two reviewers assessed each one half of the SRs. SRs were randomized to the two reviewers. In addition to the 11 items of AMSTAR, we added an additional item dealing with multiple comparisons across primary studies. We were already aware of this problem from prior publications on the same topic. However, this problem can be assumed to be topic-related and does not apply to SRs of NRS in general. We decided to exclude this item from the analysis against the background of this study.

In accordance with the AMSTAR developers, we define a NRS as a study with an observational design [13].

### Reliability, feasibility and applicability

We followed the COSMIN initiative where reliability is defined as “the degree to which the measurement is free from measurement error” [14]. Feasibility is interested in whether the measurement can be applied easily, given constraints of time, money, and interpretability according to the OMERACT initiative [15]. There is no well-accepted definition of “applicability” in our context. We have chosen the term “applicability” to give a direct to answer to the question whether AMSTAR can be applied to SRs of NRS.

We calculated Cohen’s kappa as a measure of reliability for each item (“yes” scores vs. any other scores) [16]. Kappa values of less than 0 were rated as less than chance agreement; 0.01–0.20, slight agreement; 0.21–0.40, fair agreement; 0.41–0.60, moderate agreement; 0.61–0.80, substantial agreement; and 0.81–0.99, almost perfect agreement [17]. SPSS (version 21; SPSS Inc., Chicago, IL, USA) was used to analyze the data, and the results were expressed as means and 95% confidence intervals (CI) unless otherwise noted. Furthermore, we recorded the time to complete scoring. We also listed any case where scoring was difficult or impossible. Based on these findings we investigate the applicability of AMSTAR to SR of NRS by reporting our experience on an item-by-item basis. In particularly, we highlight differences when applying AMSTAR for SRs of RCTs compared with SRs of NRS.

## Results

### Reliability and feasibility

The inter-rater reliability was high, as indicated by an overall kappa of 0.76 (95% CI: 0.76, 0.77) (range: 0.53 - 1.0). However, items 8 (scientific quality used in formulating conclusions), 9 (appropriate method to combine studies), and 11 (conflicts of interest) scored moderate at 0.57, 0.53, and 0.58, respectively (Table 1). Highest kappa values scoring >0.90 were found for item 2 (double data selection and data extraction), 6 (study characteristics), and 10 (publication bias).

**Table 1:** **Inter-rater reliability**

Items	Kappa (95% CI)
1. Was an “*a priori*” design provided?	0.65 (0.54, 0.76)
2. Was there duplicate study selection and data extraction?	0.94 (0.91, 0.96)
3. Was a comprehensive literature search performed?	0.67 (0.62, 0.72)
4. Was the status of publication (*i.e.*, grey literature) used as an inclusion criterion?	0.85 (0.81, 0.88)
5. Was a list of studies (included and excluded) provided?	0.75 (0.71, 0.79)
6. Were the characteristics of the included studies provided?	0.91 (0.88, 0.94)
7. Was the scientific quality of the included studies assessed and documented?	0.61 (0.56, 0.66)
8. Was the scientific quality of the included studies used appropriately in formulating conclusions?	0.57 (0.51, 0.62)
9. Were the methods used to combine the findings of studies appropriate?	0.53 (0.48, 0.57)
10. Was the likelihood of publication bias assessed?	1
11. Were potential conflicts of interest included?	0.58 (0.54, 0.63)

CI Confidence interval.

There was much difference between the two pairs of reviewers. The inter-rater reliability for pair 1 had an overall kappa of 0.58 (95% CI: 0.57, 0.58), while the kappa for pair 2 had an overall kappa of 0.99 (95% CI: 0.98, 0.99).

AMSTAR proved to be easily applicable to SRs of NRS, each review taking 5–10 minutes to complete with no difference between the three reviewers.

### Applicability

#### Item 1: was an “a priori” design provided?

In general, there should be no difference with respect to this item. However, it might be more difficult to define relevant study designs for inclusion, as the definition of NRS allows for more than one study design (e.g. cohort study, case–control study, controlled before-after study).

#### Item 2: was there duplicate study selection and data extraction?

There are no differences with respect to this item.

#### Item 3: was a comprehensive literature search performed?

There are no differences with respect to this item.

#### Item 4: was the status of publication (i.e., grey literature) used as an inclusion criterion?

There are no differences with respect to this item.

#### Item 5: was a list of studies (included and excluded) provided?

There are no differences with respect to this item.

#### Item 6: were the characteristics of the included studies provided?

We faced some problems assessing this item. There were some discussions between the reviewers about the sufficient level of detail with respect to the nature of our included SRs. For example, a high quality SR on the volume-outcome relationship in pancreatic surgery provided characteristics on study period, cut-off values for volume categories, number of patients, country of origin, data source, data type (administrative vs. clinical), case mix (adjustments for comorbidity, severity and acuity of admission) and mortality rates and/or survival rates [18]. The authors provided no data on patient characteristics, although they are explicitly mentioned in AMSTAR.

#### Item 7: was the scientific quality of the included studies assessed and documented?

It turned out to be very tricky to answer this item as there is no “gold standard” for the critical appraisal of NRS. Thus, it is difficult to state any characteristics that should be covered inevitably in assessing the methodological quality of NRS.

#### Item 8: was the scientific quality of the included studies used appropriately in formulating conclusions?

This item is very much related to item 7. Assuming that the quality of included studies has not been assessed appropriately it is meaningless to assess whether the results of the critical appraisal were used appropriately in formulating conclusions.

#### Item 9: were the methods used to combine the findings of studies appropriate?

We think that this item can be applied to SR of NRS.

#### Item 10: was the likelihood of publication bias assessed?

In general, this item can be easily applied to SRs of NRS.

#### Item 11: was the conflict of interest included?

This item can be applied to SR of NRS.

## Discussion

AMSTAR showed good psychometric properties when applied to SRs of NRS. The results of the inter-rater reliability are comparable to prior findings when AMSTAR had been applied to SRs of RCTs. There are only two remarkable differences when comparing our findings to one of the first validation studies where AMSTAR was applied by two reviewers on 30 selected SRs [7]. We yielded a much higher kappa value for item 4 (publication status): 0.85 vs. 0.38 and a much lower kappa value for item 11 (conflicts of interest) 0.58 vs. 0.92. The low kappa value for item 11 in our study can be explained by differing understandings. Although the item is clearly formulated and described, we had doubts about handling it regarding the conflict of interests of health technology agencies (HTA) as there were some HTA reports in our sample of 32 reviews. Uncertainty arose in particular whether governmental agencies had to state their conflicts of interests. As one might assume that they don’t have any, it can be questioned whether it is necessary to report this in a HTA. It took us less time to complete the AMSTAR ratings for each review as in prior studies. This is probably a result of applying AMSTAR by our research team in many projects before. However, our results should be treated cautiously. We found a huge difference for the inter-rater reliability among the two pairs of reviewers, although all three reviewers had much experience in applying AMSTAR and had worked together on several occasions. There seems to be a degree of interpretability in the items. We cannot preclude that although we have randomized the SRs to the reviewers this has an impact on our results, as the sample was small (n = 32). This remains difficult to interpret. The aforementioned validation study included only 30 SRs and there were only two reviewers present [7].

Based on our experience in applying AMSTAR to SRs of NRS, we think that AMSTAR can be applied to SRs of NRS, although there are some specific points users should take care of. We faced no problems in applying the first five items of AMSTAR, but we faced problems with respect to the remaining items. Items 6 to 9 resulted in some discussions among the reviewers. They mainly arose due to the lack of standards for NRS when compared with RCTs. Items 10 and 11 can be applied to SR of NRS. Nevertheless, we faced here some problems as well. However, we believe that these cannot be generalized to all SR of NRS, but depend on the topic of the SR.

Looking at item 6 (study characteristics), it is not completely clear, whether the problems we faced with this item were NRS specific. It might also be the case that they simply reflect the difficulty of providing detailed information of a huge number of single studies in an article where space is limited.

Item 7 (critical appraisal) mainly refers to an adequate quality assessment tool for NRS. There is no clearly recommended tool for assessing the quality of volume-outcomes studies. One could also think of volume to be a prognostic factor favoring a tool for prognostic studies [19]. The Newcastle Ottawa Scale has been recommended by a number of journals (e.g. the British Journal of Surgery). At the time of writing it was validated for the first time [20]. At the same time a research group developed and validated a tool for assessing the risk of bias in NRS. The Risk of Bias Assessment Tool for Nonrandomized Studies (RoBANS) showed moderate reliability and promising validity [21]. According to the authors, it was developed to be used for the assessment of virtually all study designs except for RCTs. It is also far from clear whether critical appraisal tools for NRS can be applied to registry-based studies. For example, questions dealing with incomplete data or missing data can’t be applied easily as registries might only incorporate data of cases with complete data. Furthermore, data quality of the registry is hardly to assess based on a journal article. Searching for secondary sources on the data quality would be necessary in many cases as there is not enough information in many registry-based studies.

In general, there is much heterogeneity in methods applied in observational studies [22]. To account for confounding and bias regression models are used often. However, it has been debated that they are not able to fully correct for all biases [23]. Understanding and assessing the quality of regression models is much more difficult when opposed to most analysis methods used in randomized controlled trials. One needs to have expertise in epidemiology, statistics or related sciences to be able to assess the methodological quality of NRS using regression models due to their complexity and variation. Discussions may also arise about the most appropriate model for a study.

Item 9 (combining findings) was very challenging for the raters. In our case, many SR also performed a meta-analysis. It should be kept in mind that there are fundamental differences in assumptions made to meta-analyses either for RCTs or NRS. It is assumed that a RCT provides an unbiased estimate of the effect, while observational studies yield estimates of association that do not necessarily reflect the true effect mainly due to the effects of confounding and/or bias [24]. To overcome this, it has been recommended to pool bias-adjusted results for each study instead [25].

Most studies on the volume-outcome relationship treat volume as a categorical variable. Taking volume as an outcome measure can be confusing, as the number of procedures performed can classify the same hospital as low volume or high volume, depending on the geographical area. To overcome this, meta-analyses mostly pooled the effect sizes of single studies when opposing the highest volume category to the lowest volume category. This is also a problem with respect to item 10 (publication bias). In our case, assessing this item was confusing. This was mainly due to the fact of non-comparable effect sizes as they originate from comparisons of various volume categories making them hardly comparable. A visual inspection of the funnel plot will be misleading under these circumstances. This introduces the problem that one might judge this item to be fulfilled if the authors assess publication bias, although this should not have been done for methodological reasons. It should be kept in mind that publication bias is supposed to be higher in observational studies than in RCTs [26]. Furthermore, we suspect that there is a kind of “hidden” publication bias because of registry data. If registry data are available they must not be necessarily analyzed and published. Registry data may also introduce the problem of double-counting when persons who take part in a study are also included in a registry leading to double-analyses of one case.

Although item 11 (conflicts of interest) can be applied to SRs of NRS it might be questioned here as well, whether conflict of interest is not of much more importance for randomized trials than for NRS. As RCTs are considered to be the gold standard in assessing the efficacy of pharmaceuticals, we assume that they are more often industry-driven than in the case of studies on the volume-outcome relationship in surgery.

When talking about NRS, we should notice that study designs are often ill-defined. Classifying study designs may lead to a surprisingly low agreement [27]. Even questions such as “Was there a single cohort?” or “Was there a comparison?” turned out to be difficult to answer. Thus, a clearer concept of NRS should be presented to avoid confusions. For instance, the taxonomy for studies of interventions and exposures presented by Hartling et al. don’t use the term NRS [27]. Instead they define non-randomized trials (NRTs) as “a study in which individuals or groups of individuals (e.g. community, classroom) are assigned to the intervention or control by a method that is not random (e.g. date of birth, date of admission, judgement of the investigator). Individuals or groups are followed prospectively to assess differences in the outcome(s) of interest. The unit of analysis is the individual or the group, as appropriate.” Furthermore, beside of the known “classical” observational studies such as cohort studies or case–control studies, there are a number of additional study designs. The taxonomy presented by Hartling et al. differentiate between RCTs, NRTs, prospective/retrospective cohort studies, interrupted time series with/without comparison group, (controlled) before-after-studies, (nested) case–control studies, non-concurrent cohort studies, cross-sectional studies and non-comparative studies. The Cochrane Handbook even distinguish more study designs [28]. Our analyzed SRs included predominantly cohort studies. Thus, our conclusions relate primarily to SRs of cohort studies. We are not sure whether our findings can be generalized to SRs of the above mentioned study designs. Developers of tools for assessing the quality of SRs of NRS should clearly describe their concept of NRS. This may also include a distinction between review types (e.g. intervention review or prognostic review). Keeping the variety of study designs in mind (as described above) the concept of NRS seems to be not more than a differentiation from the concept of a RCT. Developing a tool for SRs of NRS might be helpful when compared to the current situation where we only have a validated tool for SRs of RCTs, but it may neglect specific study design characteristics. It should be questioned whether the concept of NRS is too broad in this context.

## Conclusion

AMSTAR can be applied to SR of NRS, albeit we noticed some problems. Nevertheless, it seems that all items can be applied generally, although some revisions and extensions might be helpful. This is more relevant to the explanations of each item than for the formulation of them. Future studies should also focus on the psychometric properties of AMSTAR for SR of NRS. These should also try to include more than one pair of raters. Although we were able to show reliability for AMSTAR for SR of NRS, we did not investigate validity. However, there can’t be validity without reliability, while there can be reliability without validity.
